# HCV affects K_ATP_ channels through GnT-IVa-mediated N-glycosylation of GLUT2 on the surface of pancreatic β-cells leading to impaired insulin secretion

**DOI:** 10.1007/s12020-023-03589-z

**Published:** 2023-11-14

**Authors:** Ben Niu, Lijing Ma, Lixuan Yao, Yating Zhang, Heng Su

**Affiliations:** 1 Department of Endocrinology and Metabolism, The First People’s Hospital of Yunnan Province, The Affiliated Hospital of Kunming University of Science and Technology, Kunming, 650032 Yunnan China; 2 Department of Nephrology, Bao Ji People’s Hospital, Baoji, 721000 Shaanxi China

**Keywords:** Hepatitis C virus, GnT-IVa, GLUT2, N-glycosylation, K_ATP_, Insulin

## Abstract

**Purpose:**

To explore the mechanism of insulin secretion dysfunction in pancreatic beta cells induced by N-glycosylation mediated by an infection from the hepatitis C virus (HCV).

**Methods:**

Min6 cell models infected with HCV and stimulated with glucose were constructed. Meanwhile, an HCV-infected animal model and a type 2 diabetes mellitus (T2DM) rat model were constructed. Glucose uptake in the Min6 cells was detected, and insulin secretion was detected by ELISA. Flow cytometry, immunofluorescence staining, Western blotting, RT-qPCR, and lectin blotting were used to detect the expression levels of related proteins and mRNA, as well as the level of N-glycosylation. HE staining was used to observe the pathological changes in the pancreatic tissue, and an oral glucose tolerance test (OGTT) was used to evaluate the glucose tolerance of the rats.

**Results:**

Compared with the NC group, the expression levels of GnT-IVa, GLUT2, galectin-9, and voltage-dependent calcium channel 1.2 (Cav1.2) were significantly downregulated in the HCV-infected group. The ATP-sensitive potassium channel (K_ATP_) component proteins SUR1 and Kir6.2 were significantly upregulated, while intracellular glucose intake and insulin secretion decreased, N-glycosylation levels and ATP levels significantly decreased, and the overexpression of GnT-IVa reversed the effect of the HCV infection. However, treatment with the glycosylation inhibitor kifunensine (KIF) or the K_ATP_ channel activator diazine (Dia) reversed the effects of the overexpression of GnT-IVa. In the animal experiments, HE staining revealed serious pathological injuries in the pancreatic tissue of the HCV-infected rats, with decreased glucose tolerance and glycosylation levels, decreased insulin secretion, downregulated expression of GnT-IVa, GLUT2, and Cav1.2, and upregulated expression of SUR1 and Kir6.2. The overexpression treatment of GnT-IVa or the K_ATP_ channel antagonist miglinide reversed the effects of HCV.

**Conclusion:**

HCV infection inhibits GLUT2 N-glycosylation on the pancreatic β cell surface by downregulating the expression of GnT-IVa and then activates the K_ATP_ pathway, which ultimately leads to disturbances in insulin secretion.

## Introduction

As a spherical, single-stranded positive-strand RNA virus less than 80 nm in diameter, the hepatitis C virus (HCV) belongs to the Flaviviridae family. HCV is a hepatotropic virus that mainly acts on the liver. As an important endocrine gland in vivo, liver damage can lead to a variety of metabolic diseases, such as impaired glucose tolerance and insulin resistance [1]. HCV infection is linked to an increased risk of type 2 diabetes mellitus (T2DM), and HCV and diabetes mellitus have a clinical correlation [2]. The results of one study show that the probability of an HCV infection complicated with diabetes is approximately 21–50%. Subsequently, Shintani et al. [3] found that HCV-infected persons were prone to T2DM and that there is a complex relationship between HCV infections and insulin [4, 5]. Therefore, it is important to explore the mechanism of insulin secretion disorders resulting from HCV infections to reduce the damage of HCV to multiple human organs.

It is well known that the main pathogenic mechanism of diabetes mellitus includes functional defects in islet beta cells, which can lead to insufficient insulin secretion or poor interaction with insulin receptor substrates, resulting in glucose metabolism disorders and eventually hyperglycemia. In the pathogenesis of diabetes, pancreatic beta cells have important functions [6]. In addition, Negro et al. [7] showed that HCV can replicate not only in the liver but also in the pancreas, causing damage to islet beta cells, causing functional defects and interfering with insulin secretion. Insulin secretion is related to glucose uptake. Glucose uptake in islet β cells mainly depends on the expression levels and activity of glucose transporters (GLUT) [8]. GLUT2, a member of the GLUT family, is a glycosylated cell membrane protein. Glycosylation modification of GLUT2 is essential to maintain normal physiological function, regulate glucose homeostasis, and prevent the formation of T2DM [9]. GlcNAcT-IVa glycosyltransferase (GnT-IVa) is a key molecule in the regulation of GLUT2 glycosylation. It has been found that the knockout of GnT-IVa can reduce the half-life of GLUT2 on the surface of cell membranes, resulting in GLUT2 internalization and transport to the endosomes and lysosomes for degradation. It is hypothesized that GnT-IVa has an important function in regulating GLUT2 glycosylation and allowing GLUT2 to reside on the cell surface [10].

It was demonstrated that adenosine triphosphate-sensitive potassium (K_ATP_) channels have important functions in regulating insulin secretion [11], which is mainly regulated by extracellular glucose concentrations in islet beta cells. When glucose concentrations increase, GLUT2 on the surface of islet beta cells internalizes glucose into the cells and metabolizes it to produce a large amount of adenosine triphosphate (ATP). The depolarization of the cell membrane is caused by an increase in the ATP/ADP ratio which closes the K_ATP_ channel. Thus, the voltage-dependent calcium channels open, and Ca^2+^influx and insulin release occur [12]. Kasai et al. [13] reported that HCV infection downregulates the surface expression of GLUT2 in hepatocytes, thus reducing glucose uptake by hepatocytes. However, it has not been reported whether the dysfunction of insulin secretion induced by HCV infection is caused by the downregulation of the GnT-IVa-mediated N-glycosylation of GLUT2 on the surface of pancreatic beta cells affecting K_ATP_ channels. The objective of this study was to establish a rat model of T2DM and HCV infection to explore and verify these questions at the cellular and animal levels.

## Methods and materials

### Cell origin and culture conditions

The mouse pancreatic beta cells (Min6) and human hepatoma cells (Huh7.5.1) were derived from the ATCC cell bank. We used RPMI 1640 medium to culture the Min6 cells. Meanwhile, we used DMEM, with 10% FBS and 1% antibiotic (penicillin and streptomycin), to culture the Huh7.5.1 cells. The parameters of the cell incubator were 37 °C and 5% CO_2_, a microscope was used to observe the morphology of the cells, and the culture medium was changed and passaged routinely. When the cell density reached 80%, the follow-up experiment was carried out.

### Cell transfection

In the logarithmic growth phase, the cells were digested with 0.25% trypsin solution and seeded in a 24-well plate. The parameters of the cell incubator were 37 °C and 5% CO_2_, and a microscope was used to observe the morphology of the cells. The oe-GnT-IVa was transfected with a Lipofectamine ® 3000 reagent and used for subsequent experiments after the successful transfection.

### HCV infection and glucose handling

HCV infection: the pJFH1 obtained by in vitro transcription was incubated with the human hepatoma cell line Huh7.5.1 at 37 °C in a 5% CO_2_ incubator for 15 days. The supernatant was collected after centrifugation (3000 rpm, 5 min), a high-purity viral RNA kit (Roche, Switzerland) was used for quantification by agarose gel electrophoresis, and different viral loads of HCV (0.02 × 10^6^ IU/mL, 0.05 × 10^6^ IU/mL, and 0.08 × 10^6^ IU/mL) were used to treat the Min6 cells. In the KRBH buffer containing 0. 1% BSA, the Min6 cells were incubated for 60 min to carry out the glucose treatment, and then 5 mM, 10 mM, 20 mM, and 40 mM glucose were added to stimulate the Min6 cells.

### Flow cytometry

The cells were digested and passaged with trypsin and inoculated into Petri dishes overnight. Then GLUT2 antibody (1:500, Abcam, UK) was incubated with the cells for 60 min on ice. After washing, the cells were incubated with FITC-labeled goat anti-rabbit IgG (1:200, Abcam, UK) for 60 min on ice. Detection was performed by flow cytometry (Thermo Fisher, USA). Images were processed and analyzed using FlowJo X software.

### Laboratory animals

Eight-week-old male SD rats weighing 180–200 g were obtained from Kunming Medical University. The experimental protocol was approved by the Animal Ethics Committee of Kunming University of Science and Technology and met the requirements of the National Institutes of Health Guidelines for the Care of Laboratory Animals. The conditions under which the rats were fed adaptively for 7 days were 22–26 °C, relative humidity of 52–58%, and 12 h of light. The rats were randomly divided into the Control group, T2DM group, HCV infection group, HCV + oe-GnT-IVa group, and HCV + mitiglinide group. In the T2DM group, we used high-fat and high-sugar food to feed the rats for 4 weeks, and then a 1% streptozotocin (STZ) solution was injected at a dose of 35 mg/kg. After three days, all the rats fasted for 12 h and were free to drink water. Fasting blood glucose of rats was detected by glucose meter. If the blood glucose level exceeded 16.7 mmol/L, the rats were considered to meet the standard of T2DM model, and the relevant experiments were carried out after continued feeding for 4 weeks. The rats in the HCV-infected group were treated with the same conditions as in a previous study [14]. The rats were injected intravenously with 10^5^–10^7^ VGE of RHV-rn1. The rats in the HCV + oe-GnT-IVa group were injected with 100 μL of the GnT-IVa overexpression plasmid via the tail vein, and in the Control group, the rats were injected with the same dose with saline. The rats in the HCV + mitiglinide group were given mitiglinide (10 mg/kg) by gavage once a day.

### Real-time quantitative PCR (RT-qPCR)

An RNA kit was used to obtain total RNA. Care was taken to avoid RNA degradation and contamination during extraction. The samples were frozen in liquid nitrogen, and then TRIzol reagent was added to isolate the total RNA. Fluorescence quantitative PCR was performed using cDNA with the following reaction conditions: predenaturation at 95 °C for 20 s; then, the amplification cycle was carried out at 95 °C for 1 s and 60 °C for 20 s, and there were 40 cycles in this stage. Then, a dissolution curve analysis stage was performed, wherein the temperature of the dissolution curve was set to 60–95 °C, and each sample was provided with three duplicate wells. Using GAPDH as a control, the level of the target product relative to the internal control was expressed as 2^−ΔΔCt^. The primer sequences are shown in Table 1.

**Table 1:** Primer sequences

Gene	Primer	Sequence (5“-’‘)
CLUT2	For	5′-GAAGACAAGATCACCGGAACCTTGG-3′
	Rev	5′-GGTCATCCAGTGGAACACCCAAAA-3′
GnT-IVa	For	5′-TGAAGCCATTGCTTCTCAAGGTCC-3′
	Rev	5′-GGCCCAAACAGCTGAGTTCTGAAT-3′
galectin-9	For	5′-TCTGGGACTATTCAAGGAGGTC-3′
	Rev	5′-CCATCTTCAAACCGAGGGTTG-3′
SUR1	For	5′-ACCAAGGTGTCCTCAACAACGGCT-3′
	Rev	5′-ACCAAGGTGTCCTCAACAACGGCT-3′
Kir6.2	For	5′-TGCTGTCCCGAAAGGGCATTATC-3′
	Rev	5′-TGCAGTTGCCTTTCTTGGACACG-3′
Cav1.2	For	5’-AGACGCTATGGGCTATGA-3’
	Rev	5’-AACACCGAGAACCAGATTTA-3’
HCV	For	5’-CGGACGTAGCAGTGCTCACTTC-3’
	Rev	5’-TGATGAGCTGGCCAAGGAGG-3'
GAPDH	For	5′-GGAGTCCACTGGTGTCTTCA-3′
	Rev	5′-GGGAACTGAGCAATTGGTGG-3′

### Western blot

The tissue and cells were first frozen and ground in liquid nitrogen, and then RIPA lysis solution was added to lyse the samples. The concentration was detected by a BCA reaction kit. After quantitative analysis, the total protein was denatured. SDS-Page gel was used for the electrophoresis, the electrophoresis apparatus (Bio-RAD, USA) was adjusted to 120 V, a PVDF membrane (Millipore, USA) was used for the membrane transfer, and skim milk (Sigma, USA) was used for blocking. Prediluted primary antibodies (Abcam, UK) were GnT-IVa (1:1000), GLUT2 (1:500), galectin-9 (1:500), insulin (1:1000), SUR1 (1:1000), Kir6.2 (1:1000), and Cav1.2 (1: 1,000) at 4 °C overnight. The next day, goat anti-mouse antibody (1:2000; Abcam, UK) or goat anti-rabbit antibody was incubated for 1 h with slow shaking at 25 °C. An ECL chemiluminescence solution was used for the development, a chemiluminescence instrument was used for the exposure and observation, and ImageJ was used for the protein band analysis.

### Lectin blot

The plant agglutinin, sealing liquid, and the like were prepared proportionally. Immediately after electrotransfer, the mixture was stirred at 4 °C and incubated with a BSA-blocking solution overnight. At 25 °C, the membranes were incubated with lectin for 120 min. We subsequently washed the membrane three times (10 min each) with TTBS and incubated it for 60 min with 0.2 g/ml streptavidin conjugated with horseradish peroxidase in the blocking solution. The membrane was washed again with TTBS in the same way. Bound lectins were visualized by a chemiluminescence detection system and recorded on CL-xPosure films.

### Immunofluorescence staining

The cells were grown on glass covers overnight to prepare the cell slides, which were then treated with 4% paraformaldehyde. Then, Triton X-100 (0.2%) was used to permeabilize the cells, and 1% bovine serum albumin was used to block it. Then, at 4 °C, the cells were cultured with primary antibodies GnT-IVa (M-71, 1:400, Santa Cruz Biotechnology, CA, USA) for 12 h. After that, we used PBS to wash the samples and then incubated them with fluorescently labeled secondary antibodies (1:1000, ab150077, Abcam, UK) for 1 h in the dark. Finally, DAPI (Invitrogen, CA, USA) was added for counterstaining. The results were observed using a fluorescence microscope.

### GST pull-down experiment

A sequence encoding GnT-IVa was cloned into a carrier plasmid with a Myc tag, a sequence encoding GLUT2 was cloned into a carrier plasmid with a GST tag, the plasmids were transfected into Escherichia coli, and then GST and Myc-GnT-IVa fusion proteins were fixed in glutathione-agarose beads. After incubation at 4 °C for 60 min with gentle shaking and 3 washes, the GST-GLUT2 fusion protein was added to the immobilized GST, and the two fusion proteins were incubated in a rotary incubator. An ECL chemiluminescence solution was used for the development, a chemiluminescence instrument was used for the exposure and observation, and ImageJ was used for the protein band analysis.

### ELISA

In the cell experiments, the supernatant of the cell culture of each group was taken. In the animal experiment, the blood samples from the rats were obtained by collecting blood from the retroorbital venous plexus. To do this, the rats were fixed, a thumb and index finger gently pressed on both sides of the neck causing congestion of the orbital vein, and then a needle was inserted from the inner corner of the eye at an angle of 45° and rotated downward to make the blood flow out, and then the rat serum was separated. Insulin concentrations in the rat serum and the cell culture supernatant were determined using an ELISA kit (MIBIO, Shanghai, China).

### Hematoxylin-eosin staining

For the HE staining, we used 4% paraformaldehyde to fix the pancreatic tissues for 12 h and dehydrated them with an ethanol gradient. Then, we used paraffin to embed the tissue. HE staining was used to stain the sections (5 μm). The results were observed using a microscope.

### Oral glucose tolerance test (OGTT)

After fasting for 12 h, a 50% glucose injection (2 g/kg body weight) was administered to the rats by gavage, and blood glucose was measured at 0 min, 30 min, 60 min, and 120 min after gavage to evaluate the glucose tolerance of the rats.

### Glucose uptake assay

A glucose uptake test kit (Thermo Fisher Scientific, Cat. No.: N13195) was used to detect the glucose uptake in the Min6 cells. The cells were inoculated on 96-well plates and incubated in an incubator for 24 h. The cells were treated with glucose at 37 °C for 1 h, and then incubated with 100 μM 2-NBDG staining solution for 20 min. Untreated control cells were stained under the same conditions. Fluorescence signals were measured using fluorescence microscopy.

### ATP assay

The CellTiter-Glo™ luminescent cell viability assay kit (Promega) was used to evaluate the intracellular ATP content [15]. Briefly, the cells were seeded in 96-well plates (3 × 10^3^ cells/well) and allowed to grow for 24 h. A CellTiter-Glo reagent (50 μl) was then added directly into each well, incubated for 10 min, and then the plate was read with an enzyme marker. The ATP content was then calculated by the luminescence level of the cells.

### Mitochondrial membrane potential detection

Mitochondrial membrane potential was evaluated using the mitochondrial membrane potential detection kit (JC-1) (Beyotime, Shanghai, China). According to the kit instructions, cells were incubated with 200 μL JC-1 staining solution at 37 °C for 20 min away from light. Then the fluorescence microscope was used to observe and photograph (JC-1 monomer was green fluorescence, aggregate was red fluorescence).

### Statistical analysis

For all statistical analyses, GraphPad Prism7 was used. All experiments were divided into three parallel groups, and the experiment was repeated at least three times. The results are presented as the mean and SD value. One-way ANOVA and *t*-test were used to analyze the data and a value of *P* < 0.05 was deemed statistically significant.

## Results

### HCV infection decreased the expression of GLUT2

**Fig. 1:**
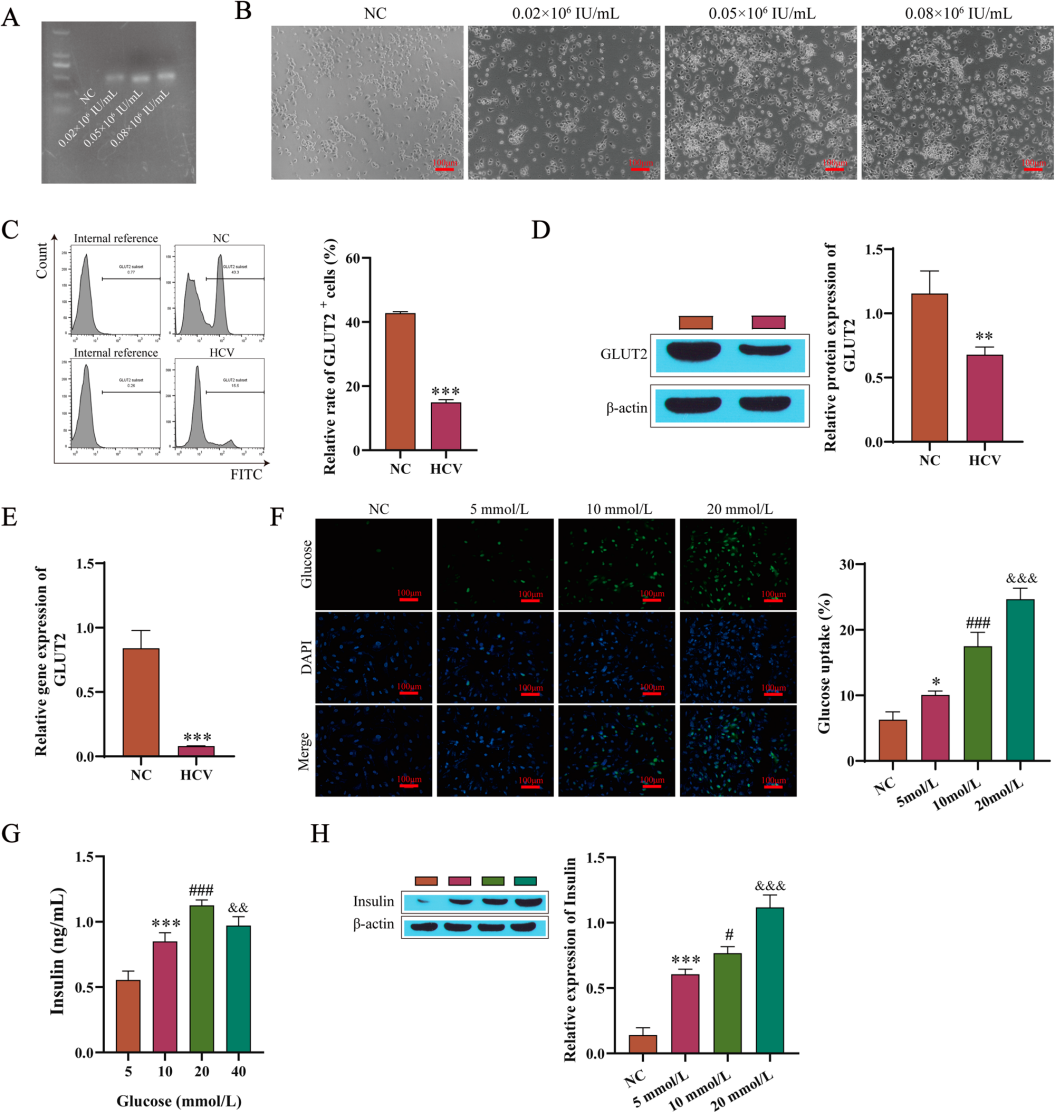
HCV infection decreases GLUT2 expression levels. **A** Agarose gel electrophoresis quantitation of the HCV. **B** Morphological changes in the Min6 cells. **C** Flow cytometry results detecting the levels of GLUT2 on the surface of the Min6 cells. **D** Western blotting results detecting the protein expression levels of GLUT2. **E** RT-qPCR results determining the mRNA expression of GLUT2. **F** The detected intracellular glucose uptake. **G** The insulin secretion was detected by ELISA. **H** Western blotting results detecting the insulin content. The 5 mM glucose group or HCV group compared with the NC group (normal Min6 cells without any treatment), *P < 0.05, **P < 0.01, ***P < 0.001; compared with the 5 mM glucose group, ^#^*P* < 0.05,^###^*P* < 0.001; and compared with the 10 mM glucose treatment group, ^&&^*P* < 0.01,^&&&^*P* < 0.001

After the treatments with the different viral loads of HCV (0.02 × 10^6^IU/mL, 0.05 × 10^6^IU/mL, and 0.08 × 10^6^IU/ml), the Min6 cells were quantified by agarose gel electrophoresis. The results showed that an infection load of 0.05 × 10^6^IU/mL worked best (Fig. 1A). Second, the death of the Min6 cells increased with increasing viral load (Fig. 1B), establishing that HCV infection causes damage to Min6 cells. As a glycosylated cell membrane protein, GLUT2 has a key function in maintaining the normal physiological function and glucose homeostasis of pancreatic β cells [16]. In this study, we used flow cytometry (Fig. 1C) to detect the expression of GLUT2 on the surface of Min6 cells, Western blotting (Fig. 1D) to detect the expression levels of the GLUT2 protein, and RT-qPCR (Fig. 1E) to detect GLUT2 mRNA levels. On the surface of the cells, the mRNA and protein levels of GLUT2 in the HCV infection group were significantly lower. The intracellular glucose uptake (Fig. 1F), insulin secretion (Fig. 1G), and insulin content (Fig. 1H) were detected for the cells stimulated with different concentrations of glucose. The intracellular glucose uptake, insulin secretion, and insulin content increased gradually with the glucose concentration, and the results of the insulin secretion detected by ELISA showed that the insulin secretion decreased when the concentration was 40 mM compared with 20 mM. Therefore, 20 mM glucose was selected.

### HCV infection inhibits the N-glycosylation of GLUT2

**Fig. 2:**
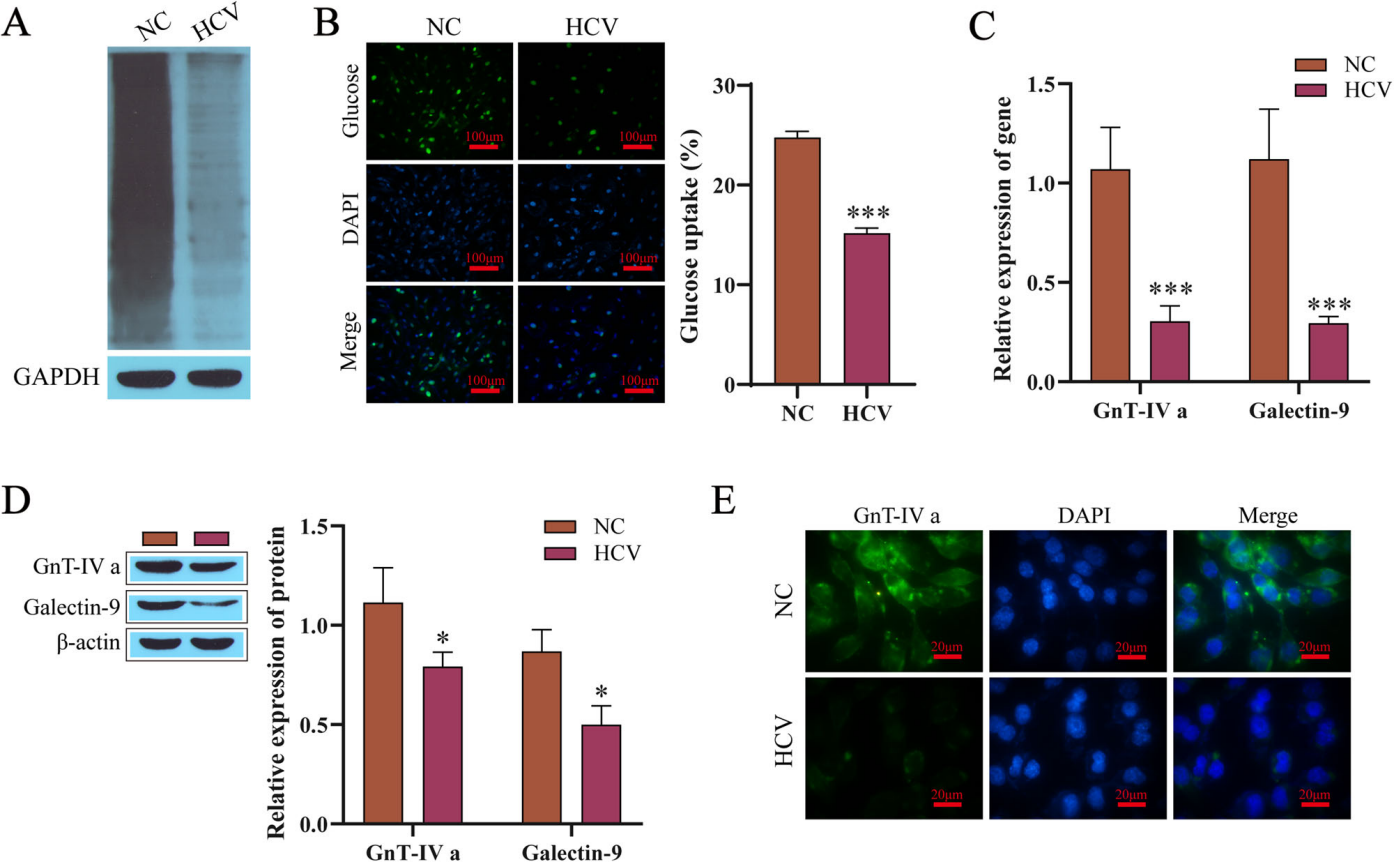
HCV infection inhibits the N-glycosylation level of GLUT2. **A** The lectin blotting results detecting the levels of N-glycosylation. **B** The intracellular glucose uptake. **C** RT-qPCR results detecting the mRNA levels of GnT-IVa and galectin-9. **D** Western blotting results detecting the protein levels of GnT-IVa and galectin-9. **E** Immunofluorescence results detecting GnT-IVa. NC: The normal Min6 cells treated with 20 mmol/L glucose. Compared with the NC group, *P < 0.05, ***P < 0.001

To explore the influence of HCV infection on the N-glycosylation levels of GLUT2, lectin blotting was used to detect the level of N-glycosylation after stimulating the NC group and HCV-infected group with 20 mM glucose. The N-glycation level of the HCV-infected group decreased (Fig. 2A). In the HCV-infected group, further detection of the glucose uptake by the cells showed that glucose uptake was reduced (Fig. 2B). GnT-IVa is a key molecule for glycosylation, and galectin-9 can form a glycosyl-lectin grid with glycosylated proteins, which affects the residence time of glycosylated proteins on the cell membrane [17]. The expression of GnT-IVa and galectin-9 (Fig. 2C, D) in the HCV-infected cells was downregulated. Through immunofluorescence detection, we found that GnT-IVa levels in the cells of the HCV-infected group were lower (Fig. 2E).

### HCV infection inhibits GLUT2 N-glycosylation by downregulating GnT-IVa

**Fig. 3:**
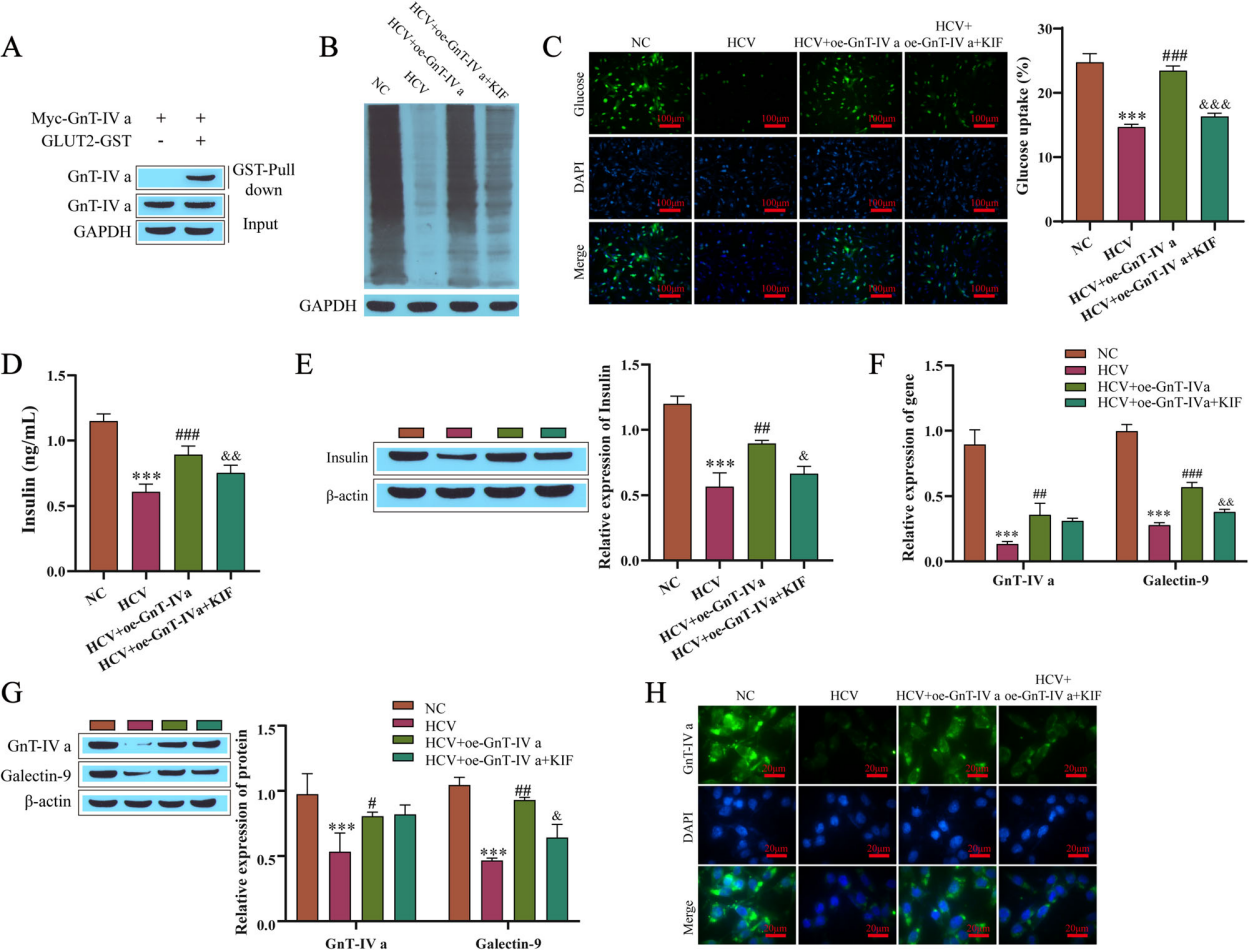
HCV inhibits the N-glycosylation of GLUT2 by downregulating the levels of GnT-IVa. **A** GST pull-down assay results determining the binding relationship between GnT-IVa and GLUT2. **B** Lectin blotting results for detecting the level of N-glycosylation. **C** Intracellular glucose uptake levels were detected. **D** Insulin secretion was detected by ELISA. **E** Western blotting results detecting the insulin content. **F** RT-qPCR results detecting the mRNA levels of GnT-IVa and galectin-9. **G** Western blotting results detecting GnT-IVa and galectin-9. **H** Immunofluorescence results detecting GnT-IVa. Results of the treatment groups compared with the NC group (normal Min6 cells treated with 20 mmol/L glucose), ***P < 0.001; compared with the HCV group, ^#^*P* < 0.05, ^##^*P* < 0.01, ^###^*P* < 0.001; compared with the HCV + oe-GnT-IVa group, ^&^*P* < 0.05, ^&&^*P* < 0.01, ^&&&^*P* ＜ 0.001

We have shown that HCV infection affects the N-glycosylation of GLUT2 and the expression of GnT-IVa. Next, we will explore whether HCV infection regulates the N-glycosylation of GLUT2 through GnT-IVa. First, the binding relationship between GnT-IVa and GLUT2 was verified by a GST pull-down assay, and the results showed that GnT-IVa and GLUT2 interacted (Fig. 3A). Next, to verify that HCV infection affects glycosylation levels through GnT-IVa, we transfected oe-GnT-IVa into cells and treated them with the glycosylation inhibitor kifunensine (KIF). Compared with the NC group, the N-glycosylation level of the HCV-infected group decreased. Compared with the HCV-infected group, the N-glycosylation level of the cells overexpressing GnT-IVa increased. However, treatment with the glycosylation inhibitor KIF attenuated the effect of oe-GnT-IVa (Fig. 3B). A glucose uptake assay showed that the glucose uptake of the cells decreased after the HCV infection, and it increased after being transfected with oe-GnT-IVa compared with the cells infected with HCV, and the KIF treatment weakened the effect of oe-GnT-IVa (Fig. 3C). The results of the insulin secretion and content detection showed that the HCV infection inhibited the secretion and content of insulin, and the secretion levels and content of insulin increased after the transfection of oe-GnT-IVa compared with the HCV infection group, and the influence of oe-GnT-IVa was weakened by the KIF treatment (Fig. 3D–E). For GnT-IVa and galectin-9, HCV infection downregulated the levels of GnT-IVa and galectin-9 mRNA and protein. The levels of GnT-IVa and galectin-9 were upregulated after the transfection with oe-GnT-IVa compared to the HCV infection group, and the KIF treatment attenuated the effect of oe-GnT-IVa (Fig. 3F–G). The same was true for further detection of the GnT-IVa expression levels by immunofluorescence staining (Fig. 3H).

### Effects of the N-glycosylation of GLUT2 on the K _ATP_ channel

**Fig. 4:**
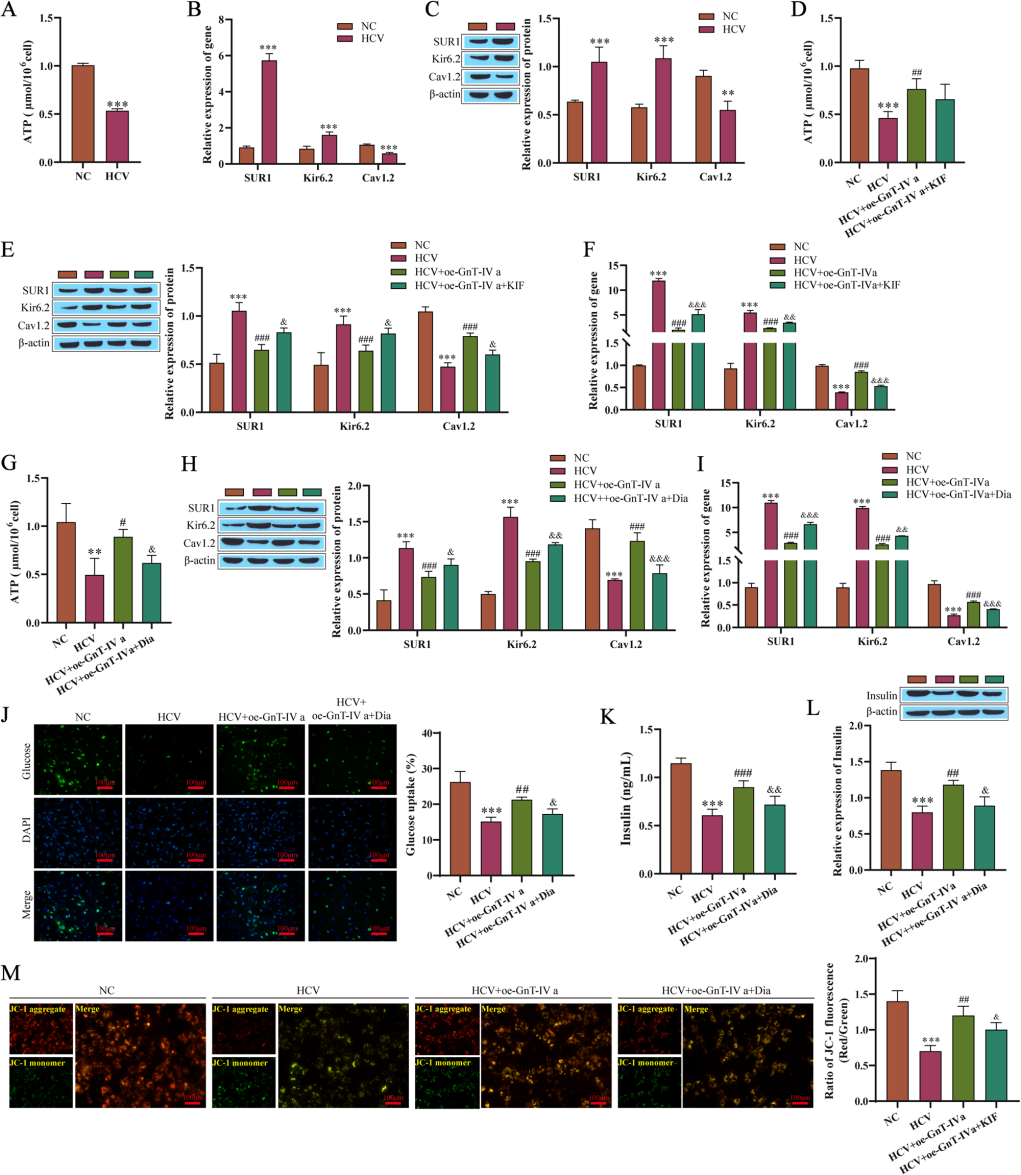
Effect of N-glycosylation of GLUT2 on the K_ATP_ channel. **A** The ATP level results. **B** RT-qPCR results detecting SUR1, Kir6.2, and Cav1.2. **C** Western blotting results detecting SUR1, Kir6.2, and Cav1.2. **D** The ATP level results. **E** Western blotting results detecting SUR1, Kir6.2, and Cav1.2. **F** The expressions of SUR1, Kir6.2, and Cav1.2 were detected by RT-qPCR. **G** The results for the levels of ATP. **H** Western blotting results detecting SUR1, Kir6.2, and Cav1.2. **I** RT-qPCR results detecting the levels of SUR1, Kir6.2, and Cav1.2. **J**, **K** Intracellular glucose uptake and insulin secretion results measured by kits. **L** Western blotting results detecting the insulin content. **M** Mitochondrial membrane potential was detected with JC-1 kit. Compared with the NC group (normal Min6 cells treated with 20 mmol/L glucose), **P < 0.01, ***P < 0.001; Compared with the HCV group, ^#^*P* < 0.05, ^##^*P* < 0.01, ^###^*P* < 0.001; Compared with the HCV + oe-GnT-IVa group, ^&^*P* < 0.05, ^&&^*P* < 0.01, ^&&&^*P* < 0.001

The K_ATP_ channel has an important function in insulin secretion in pancreatic β cells, which is regulated by intracellular concentrations of ATP [18]. We first examined the intracellular ATP levels and found that the intracellular ATP level decreased in the HCV-infected group (Fig. 4A). The mRNA levels of the K_ATP_ channel component proteins SUR1 and Kir6.2 and voltage-dependent calcium channel 1.2 (Cav1.2) were further detected by RT-qPCR and Western blotting. The mRNA and protein levels of SUR1 and Kir6.2 in the HCV-infected cells significantly increased, and the level of Cav1.2 mRNA and the expression of Cav1.2 protein were lower (Fig. 4B-C). In the above results, we have demonstrated that the overexpression of GnT-IVa increased the N-glycosylation of GLUT2 in the cells. Figure 4D–F showed that, compared with the HCV infected group, after overexpression of GnT-IV a, intracellular ATP level was increased, the expression levels of SUR1 and Kir6.2 were decreased, and the expression level of Cav1.2 was significantly increased, while the KIF treatment attenuated the effect of the overexpression of GnT-IVa. This suggests an effect of the N-glycosylation of GLUT2 on the K_ATP_ channel. To further confirm the influence of K_ATP_ channels in the dysfunction of insulin secretion mediated by the N-glycosylation of GLUT2, intracellular ATP levels and the expression of SUR1, Kir6.2, and Cav1.2 were measured after the HCV infection treatment and the overexpression of GnT-IVa with the addition of K_ATP_ channel activator diazoxide (Dia). The results showed that the Dia treatment attenuated the effect of the overexpressing of GnT-IVa, consistent with the effect of KIF (Fig. 4G–I). The glucose uptake and insulin content and secretion in the HCV infection group were lower, and the GnT-IVa overexpression effectively increased the glucose uptake and insulin content and secretion. However, the activation of the K_ATP_ channel reversed the effect of oe-GnT-IVa (Fig. 4J–L). The ratio of JC-1 aggregate/monomer in cell fluorescence was used to evaluate mitochondrial membrane potential, and it was found that HCV infection significantly reduced the membrane potential, while overexpression of GnT-IVa alleviated the effect of HCV and promoted the increase of membrane potential, and the effect of overexpression of GnT-IVa was weakened after addition of Dia treatment (Fig. 4M).

### HCV affects rat pancreatic islet function by mediating the N-glycosylation of GLUT2

**Fig. 5:**
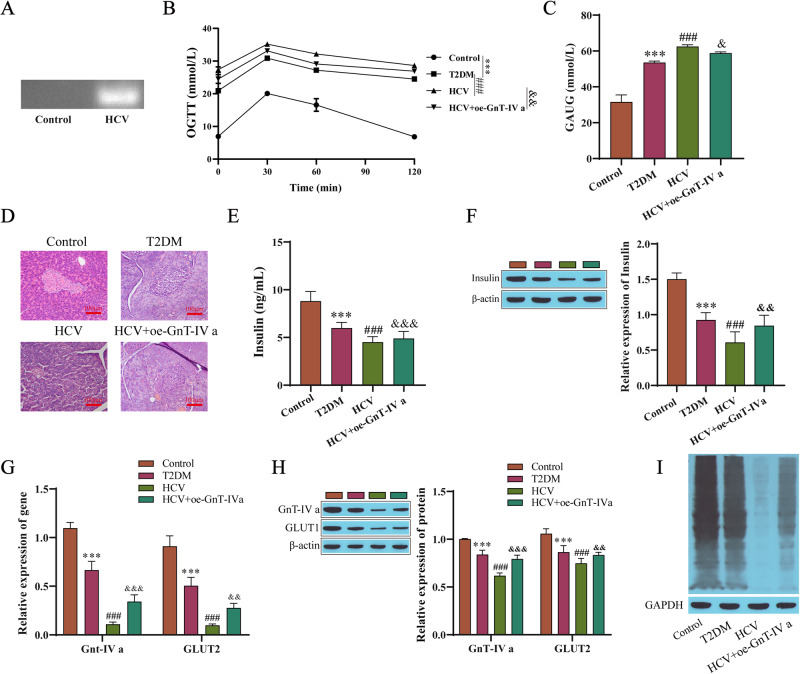
HCV affects rat islet function by mediating N-glycosylation of GLUT2. **A** Detection of HCV expression. **B** Oral testing of the rats’ glucose tolerance levels. **C** The area under the curve of blood glucose. **D** The pathological changes in the pancreatic tissue observed by HE staining. **E** ELISA results detecting the insulin secretion. **F** Western blotting results detecting the insulin content. **G** RT-qPCR results detecting GnT-IVa and GLUT2 in the rat pancreatic tissues. **H** The expression of GnT-IVa and GLUT2 were detected by Western blotting. **I** Lectin blotting results detecting the N-glycosylation levels. ***P < 0.001 compared with the control group; ^###^*P* < 0.001 compared with the T2DM group; ^&^*P* < 0.05, ^&&^*P* < 0.01, ^&&&^*P* < 0.001 compared with the HCV group

The function by which HCV infection inhibits the N-glycosylation of GLUT2 on pancreatic β cells and activates K_ATP_ channels by downregulating GnT-IVa has been demonstrated in our cell experiments mentioned above, thus we verified this mechanism in animal experiments. We used rats to carry out the experiment, and the RT-qPCR products were detected by agarose gel electrophoresis to determine whether the rats were infected with HCV. The copy number in the HCV group was significantly higher than that in the control group (Fig. 5A), indicating that a rat model of HCV infection in vivo was successful. The glucose tolerance levels of the rats in each group were evaluated by the OGTT test. The blood glucose of rats in the T2DM group increased sharply after taking glucose, peaked at 30 min to 60 min, and was still higher than the normal level two hours later, indicating successful T2DM modeling. In the control group, the blood glucose levels returned to normal 2 h after taking glucose. The OGTT curve showed that the blood glucose of rats increased to varying degrees 30 min after the intragastric administration in each group, but the blood glucose of the rats in the HCV group was higher than that in the T2DM group during the whole process, and the slope declined after 30 min and was smaller than that in the T2DM group. In the HCV group, the curve of blood glucose was larger than that in the T2DM group. In the GnT-IVa overexpression group, the blood glucose increase was smaller than that in the HCV infection group, and the slope of the curve of blood glucose decrease was larger than that in the HCV infection group after 30 min. During the whole process, the blood glucose levels in the GnT-IVa overexpression group were lower, and the area under the blood glucose curve was smaller than that of the HCV-infection group (Fig. 5B, C). HE staining was used to detect the pathological changes in the pancreatic tissues of the rats. The shape of islets in the T2DM group was irregular, the outline was not clear, and there was significant atrophy. There were exocrine glands invading the islets. The shape of the islet cells was different, the arrangement was disordered, and the number was reduced. After the HCV infection, the pathological changes in the pancreatic tissue of the rats were further worsened, and the pathological changes in the pancreatic tissue of the rats overexpressing GnT-IVa were alleviated compared with those of the HCV-infected group (Fig. 5D). ELISE results showed that the serum insulin content of the rats in the HCV infection group was lower than that in the T2DM group, while the GnT-IVa overexpression group was higher than that in the HCV infection group (Fig. 5E). In the HCV-infected group, western blotting showed that the insulin content was significantly lower than that in the T2DM group, while there was a higher insulin content in the rats overexpressing GnT-IVa (Fig. 5F). The pancreatic tissues of the rats were collected, and the levels of GnT-IVa mRNA and GLUT2 mRNA and their protein levels were detected (Fig. 5G–H). The levels of GnT-IVa and GLUT2 mRNA and the levels of GnT-IVa and GLUT2 proteins in the pancreas of the rats with the HCV infection were significantly downregulated compared with those of the rats with T2DM, while after overexpressing GnT-IVa, the expression levels in the rats were upregulated compared with those of the rats with the HCV infection. Lectin blot analysis showed that the N-glycosylation level in the HCV-infected group was lower, while the N-glycosylation level in the GnT-IVa overexpression group was higher (Fig. 5I).

### HCV promotes the progression of diabetes in rats by affecting the K_ATP_ channel

**Fig. 6:**
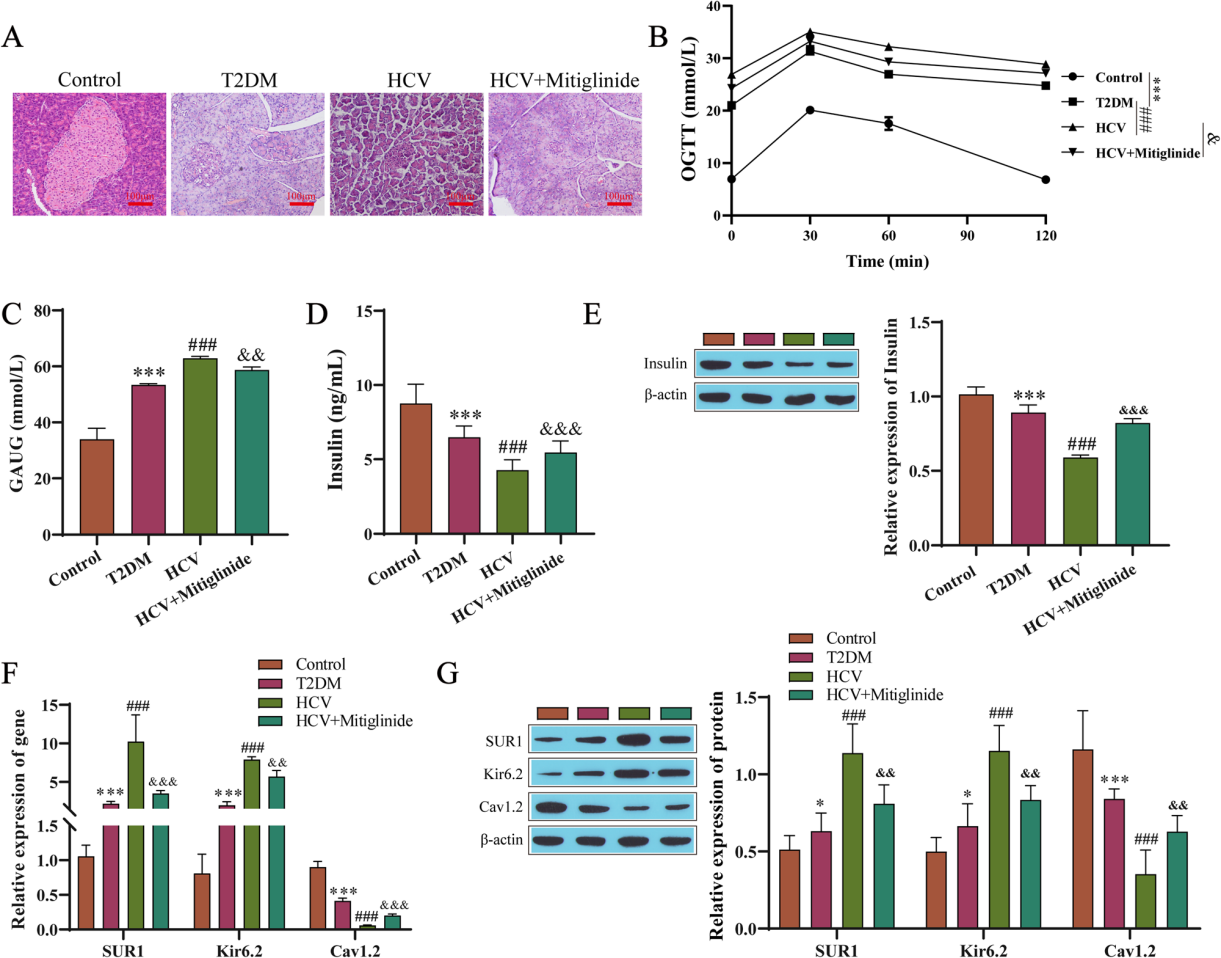
HCV promotes the progression of diabetes in rats by affecting K_ATP_ channels. **A** HE staining for pathological changes in the pancreatic tissue. **B** The tolerance test for oral glucose. **C** The area under the curve of blood glucose. **D** ELISA results detecting insulin secretion. **E** Western blotting results detecting the insulin content. **F** RT-qPCR results detecting the levels of SUR1, Kir6.2, and Cav1.2 in the rat pancreatic tissues. **G** The protein levels of SUR1, Kir6.2, and Cav1.2 in the rat pancreatic tissues were detected by Western blotting. *P < 0.05, ***P < 0.001 compared with the control group; ^###^*P* < 0.001 compared with the T2DM group; ^&^*P* < 0.05, ^&&^*P* < 0.01, ^&&&^*P* < 0.001 compared with the HCV group

Finally, we explored the impact of K_ATP_ channel activation induced by HCV on the progression of diabetes in rats. In this study, there was a control group, T2DM group, HCV infection group, and HCV infection + mitiglinide group (K_ATP_ channel antagonist: mitiglinide), and the indices of each group were detected. The shape of islets in the T2DM group was irregular, the outline was not clear, and there was significant atrophy. There were exocrine glands invading the islets, the shapes of the islet cells were different, and there was disordered arrangement and reduced islet cells. After the HCV infection, the pathological condition of the pancreatic tissue was worse than that in the T2DM group. Pathological changes in the pancreas treated with mitiglinide were alleviated compared with those in the HCV-infected group (Fig. 6A). OGTT showed that there was increased blood glucose in each group, to different degrees, 30 min after the intragastric administration, but there was higher blood glucose in the HCV group than in the T2DM group during the whole process. In the HCV group, there was a smaller slope of the curve and a larger area under the curve of blood glucose decline after 30 min than in the T2DM group. Under the mitiglinide treatment, the slope of the curve of blood glucose increase was smaller than that in the HCV-infected group, and the decrease after 30 min was larger than that in the HCV-infected group. During the whole process, there was a lower blood glucose level in the mitiglinide-treated group than in the HCV-infected group, and there was a smaller area under the blood glucose curve than in the HCV-infected group (Fig. 6B, C). The serum insulin content of the rats in the HCV infection group was lower than that in the T2DM group, while the serum insulin content of the rats in the mitiglinide-treated group was higher than that in the HCV infection group (Fig. 6D). There was a lower insulin content in the HCV-infected group than in the T2DM group, while there was a higher insulin content under the mitiglinide treatment (Fig. 6E). The mRNA and protein levels of SUR1, Kir6.2, and Cav1.2 in rat pancreatic tissues were detected by RT-qPCR (Fig. 6F) and Western blotting (Fig. 6G). The mRNA and protein levels of SUR1 and Kir6.2 were upregulated, while Cav1.2 was downregulated in the HCV infection group compared with the T2DM group. Moreover, The levels of SUR1 and Kir6.2 were downregulated in the mitiglinide-treated rats, while the protein and mRNA levels of Cav1.2 were upregulated in the mitiglinide-treated rats.

## Discussion

Hepatitis C is a common infectious disease that is prevalent worldwide and is seriously harmful to human health. According to statistics, the HCV infection rate is 3% worldwide, and the number of HCV-infected people is nearly 180 million, accounting for 2–3% of the global population [19]. HCV has become a global public health problem. Persistent HCV infection can not only cause liver damage but also cause some metabolic diseases, such as Mehta et al. [20] studies have shown that in the United States, in people over 40 years of age, the likelihood of developing T2DM after being infected with HCV was three times higher than in those without HCV. It is known that the two basic characteristics of T2DM include insulin resistance which then leads to insufficient insulin secretion. In our study, we induced a T2DM rat model using a high-fat diet and STZ. It closely mimics the natural course and metabolic characteristics of type 2 diabetes in humans [21]. The rats showed hyperglycemia and insulin resistance or deficiency, consistent with the characteristics of T2DM. Most current studies simulate T2DM in rats induced by diet and STZ [22], and low-dose STZ(35 mg/kg) injection has been shown to have metabolic syndrome replication and a relatively stable rise in blood glucose concentration after T2DM [23]. It has been proposed that HCV is directly involved in islet beta cell dysfunction, and insulin resistance has been observed in HCV-positive patients [24]. The mechanism of HCV on insulin secretion has not been fully studied. In this study, both cell and animal experiments found that HCV had a significant inhibitory effect on insulin release, and the insulin secretion of the pancreatic β cells was correlated with glucose uptake, which was closely correlated with glucose uptake and the expression levels of GLUT2.

As a member of the GLUT family, GLUT2 is specifically expressed in the liver, islet beta cells, hypothalamic glial cells, retinal, and intestinal epithelial cells [25, 26]. Kasai et al. demonstrated that HCV infection downregulates GLUT2 expression, thereby reducing glucose uptake by hepatocytes [13]. In this study, we found consistent results that the GLUT2 expression levels were significantly decreased on the surface of mouse pancreatic beta Min6 cells after the HCV infection, suggesting that HCV infection has an effect on GLUT2 expression levels in Min6 cells. As HCV-infected persons can be complicated with diabetes [27], we further used glucose to stimulate HCV-infected cells and found that after the HCV infection, glucose uptake of the cells was inhibited and insulin secretion was reduced. This may have something to do with the expression of GLUT2. The glycosylation of GLUT2 is essential to maintain its function, and GnT-IVa is a key molecule in GLUT2 glycosylation. Our study also found that GnT-IVa binds to GLUT2. It has been shown that GnT-IVa deficiency eliminates the multiantennae N-glycanes that induce rapid internalization of glycosylated GLUT2 in pancreatic beta cells. In this study, we found that the glycosylation levels of the cells decreased after the HCV infection. The expression level of GnT-IVa was also significantly reduced, resulting in a similar decrease in the glycosylation of GnT-IVa-modified GLUT2, which is a key factor in determining its ability to reside on cell membranes. In addition, galectin is also an important factor affecting the retention of glycosylated proteins on the surface of cell membranes [10]. Galectin-9 can form a glycosyl-lectin grid with glycosylated proteins. We found that the expression levels of galectin-9 protein were significantly downregulated in the HCV infection group compared with that in the NC group. Therefore, we hypothesized that the decreased expression levels of GnT-IVa affected the glycosylation level of GLUT2 and then decreased the binding of galectin-9 to GLUT2 on the cell surface, thereby reducing the glucose transport activity of the cells. Thus affecting insulin secretion. This is consistent with the conclusion reached by Ohtsubo’s research group [28]. To verify that the expression of GnT-IVa affects the glycosylation level of GLUT2 after HCV infection, we found that the glycosylation level of the cells was upregulated after the GnT-IVa overexpression treatment, and intracellular glucose uptake and insulin secretion also increased. However, treatment with the glycosylation inhibitor KIF reversed the effect of the GnT-IVa overexpression. These results suggest that HCV infection inhibits GLUT2 N-glycosylation by downregulating the expression of GnT-IVa, thereby reducing glucose uptake and inhibiting insulin secretion.

Previous studies have confirmed that the K_ATP_ channel is crucial in regulating the insulin secretion of pancreatic β cells [29]. The K_ATP_ channel is a polymeric protein complex [30] composed of four inward rectifier potassium channel (Kir6.x) subunits and four ABC protein sulfonylurea receptor (SURx) subunits. In islet beta cells, the K_ATP_ channel is a key hub for the conversion of metabolic activity into mechanical activity, which is composed of Kir6.2 and SUR1, combines cellular metabolism with membrane excitability and regulates insulin secretion. ATP is the most important physiological effector of the K_ATP_ channel. Glucose is internalized into cells for metabolism to produce large amounts of ATP. ATP sensitivity is conferred by Kir6.2 and enhanced by SUR1 [31]. The increase in the ATP/ADP ratio leads to the closure of the K_ATP_ channel, which causes the depolarization of the cell membrane and the opening of voltage-dependent calcium channels, thus causing the influx of Ca^2+^ and the release of insulin [12].

In our study, we found that after the HCV infection, the ATP levels decreased, Kir6.2 and SUR1 proteins were upregulated, and Cav1.2 proteins were downregulated, and mitochondrial membrane potential levels decreased. This indicates that K_ATP_ channels are activated and calcium channels are inhibited after HCV infection. At the same time, the overexpression of GnT-IVa influenced the activation of the K_ATP_ channel. The overexpression of GnT-IVa improved the glucose uptake of the cells, increased ATP levels, inhibited the activation of K_ATP_ channels, and then promoted insulin secretion. However, KIF, a GLUT2 glycosylation inhibitor, and Dia, a K_ATP_ channel activator, weakened the effect of the GnT-IVa overexpression. This suggests that HCV infection inhibits GLUT2 N-glycosylation by downregulating GnT-IVa, thereby activating K_ATP_ channels and blocking insulin secretion.

We further verified the results of the cell experiments at the animal level. In our study, we found that HCV-infected rats had more severe pancreatic histological damage than the T2DM rats, and their glucose tolerance decreased. It was also found that the expression of GnT-IVa in the pancreatic tissue of the rats decreased after the HCV infection, and the N-glycosylation of GLUT2 was inhibited, thus activating the K_ATP_ channel, resulting in increased blood glucose and decreased insulin secretion in the rats. The overexpression of GnT-IVa and the K_ATP_ channel antagonist mitiglinide improved the adverse outcomes after the HCV infection. This is consistent with the results of the cell experiments. At the same time, the characteristics of the Min6 cells were very similar to those of the isolated islets, suggesting that this cell line is a suitable model to study the mechanism of glucose-stimulated insulin secretion [29, 32]. In this study, we investigated the mechanism of downregulated GnT-IVa on insulin secretion only in HCV-infected murine-derived pancreatic beta Min6 cells and rat models, but not in human-derived cells. Experiments using cells from humans will help improve the clinical application of these findings.

In brief, the results of this study confirm the mechanism by which HCV infection inhibits the N-glycosylation of GLUT2 on the surface of islet beta cells by downregulating the expression of GnT-IVa, which activates K_ATP_ channels, closes voltage-dependent calcium channels, blocks Ca^2+^influx, and ultimately leads to insulin secretion disorders.

## Data Availability

The datasets used and/or analyzed during this study are available from the corresponding author upon reasonable request.
